# Accumulation of Cerebrospinal Fluid Glycerophospholipids and Sphingolipids in Cognitively Healthy Participants With Alzheimer’s Biomarkers Precedes Lipolysis in the Dementia Stage

**DOI:** 10.3389/fnins.2020.611393

**Published:** 2020-12-16

**Authors:** Alfred N. Fonteh, Abby J. Chiang, Xianghong Arakaki, Sarah P. Edminster, Michael G Harrington

**Affiliations:** Huntington Medical Research Institutes, Pasadena, CA, United States

**Keywords:** Alzheimer’s disease biomarker, amyloid, cerebrospinal fluid, dementia, glycerophospholipids, nanoparticles, sphingolipids, tau

## Abstract

Insight into lipids’ roles in Alzheimer’s disease (AD) pathophysiology is limited because brain membrane lipids have not been characterized in cognitively healthy (CH) individuals. Since age is a significant risk factor of AD, we hypothesize that aging renders the amyloid precursor protein (APP) more susceptible to abnormal processing because of deteriorating membrane lipids. To reflect brain membranes, we studied their lipid components in cerebrospinal fluid (CSF) and brain-derived CSF nanoparticle membranes. Based on CSF Aβ_42_/Tau levels established biomarkers of AD, we define a subset of CH participants with normal Aβ_42_/Tau (CH-NAT) and another group with abnormal or pathological Aβ_42_/Tau (CH-PAT). We report that glycerophospholipids are differentially metabolized in the CSF supernatant fluid and nanoparticle membrane fractions from CH-NAT, CH-PAT, and AD participants. Phosphatidylcholine molecular species from the supernatant fraction of CH-PAT were higher than in the CH-NAT and AD participants. Sphingomyelin levels in the supernatant fraction were lower in the CH-PAT and AD than in the CH-NAT group. The decrease in sphingomyelin corresponded with an increase in ceramide and dihydroceramide and an increase in the ceramide to sphingomyelin ratio in AD. In contrast to the supernatant fraction, sphingomyelin is higher in the nanoparticle fraction from the CH-PAT group, accompanied by lower ceramide and dihydroceramide and a decrease in the ratio of ceramide to sphingomyelin in CH-PAT compared with CH-NAT. On investigating the mechanism for the lipid changes in AD, we observed that phospholipase A_2_ (PLA_2_) activity was higher in the AD group than the CH groups. Paradoxically, acid and neutral sphingomyelinase (SMase) activities were lower in AD compared to the CH groups. Considering external influences on lipids, the clinical groups did not differ in their fasting blood lipids or dietary lipids, consistent with the CSF lipid changes originating from brain pathophysiology. The lipid accumulation in a prodromal AD biomarker positive stage identifies perturbation of lipid metabolism and disturbances in APP/Amyloid beta (Aβ) as early events in AD pathophysiology. Our results identify increased lipid turnover in CH participants with AD biomarkers, switching to a predominantly lipolytic state in dementia. This knowledge may be useful for targeting and testing new AD treatments.

## Introduction

Specific mutations lead inevitably to familial Alzheimer’s disease (FAD; Lacour et al., 2019; Wong et al., 2020), and gene polymorphisms are associated with late-onset Alzheimer’s disease (AD; Morelli et al., 1996; Casadei et al., 1999; Dursun et al., 2008; Du et al., 2016; Zuin et al., 2020), the most common form of AD. Some risk factors such as age, education, apolipoprotein ε4 (*ApoE*-4), hypertension, diabetes, hyperlipidemia, obesity, smoking, and head trauma have been identified, but none is necessary or sufficient to cause AD (Devanand et al., 2013; Guimas Almeida et al., 2018; Takahashi et al., 2018). Nevertheless, early AD pathology, similar to FAD, is exemplified by extracellular β–amyloid, predominantly amyloid_42_ (Aβ_42_) deposition, with intracellular neurofibrillary tangles in the later stages that include abnormally modified tau protein (Vanmechelen et al., 2001). Amyloid beta (Aβ) peptides are enzymatically cleaved from the extracellular neuronal surface of the transmembrane amyloid precursor protein (APP; Frederikse et al., 1996; Lorenzo et al., 2000). The neurotoxic Aβ_42_ aggregates and accumulates in plaques that characterize AD pathology (Perry et al., 1988; Arai et al., 2019). Apolipoprotein ε4 reduces Aβ_42_ clearance, consistent with the increased AD risk of this inherited isoform (Castellano et al., 2011; Verghese et al., 2013). In the absence of autosomal dominant mutations, it is less clear how amyloid pathology is initiated in sporadic AD. Since Aβ_42_ derives from the transmembrane APP, clues to the mechanism that links these multiple risk factors to the Aβ_42_ cascade may, therefore, be revealed in the neuronal membrane environment at the earliest pathology stage of AD.

The most significant risk factor for AD is aging, and post-mitotic neurons are especially vulnerable in aging: oxidative damage is more significant in mitochondria of neurons from the aged brain (Chakrabarti et al., 2011), mitochondrial proteins have an age-dependent loss of expression (Shevchenko et al., 2012), and lipids, the major membrane constituent, are more susceptible over time to peroxidative damage (Hulbert, 2010). Lipids, mainly in membranes, constitute about 50% of the brain’s dry mass (O’Brien and Sampson, 1965; Svennerholm et al., 1991); though their major classes have been identified, most of their molecular species remain uncharacterized. Thus, the biggest challenges to understanding the APP processing and the origins of Aβ_42_ formation are to characterize the APP’s lipid environment in CH individuals with AD biomarkers.

Of eight lipid classes (Fahy et al., 2009), glycerophospholipids (GPs) and sphingolipids (SPs) constitute diverse molecules whose perturbation may be associated with neuronal injury, neuroinflammation (Russo et al., 2018; Leishman et al., 2019), and neurodegeneration (Raszewski et al., 2016; Aufschnaiter et al., 2017; Dorninger et al., 2020; Hernandez-Diaz and Soukup, 2020), all features associated with AD pathology. Some brain lipid components have been measured in AD (Patel and Forman, 2004; Lim et al., 2014; Garner, 2010; Chew et al., 2020; Kao et al., 2020). However, studies of the prodromal pathology phase have not been undertaken, and the availability of fresh brain tissue to explore lipid chemistry is thus a major barrier. An approach to accessing brain-derived lipids through cerebrospinal fluid (CSF) was enhanced with the discovery that CSF has an abundance of membranous nanoparticles (billions per mL) and includes typical synaptic and large dense-core vesicles (Harrington et al., 2009). CSF is readily obtained in research studies *in vivo*, potentially repeatedly, allowing testing of brain-derived fluid and membrane lipids in CH individuals with or without expression of CSF biomarkers of AD.

We characterized the most abundant GPs in the CSF from an elderly, cognitively healthy (CH) population and found GPs in the CSF nanoparticle membranes differed from the supernatant fluid (Fonteh et al., 2013). Moreover, the CSF Aβ_42_ biomarker, most commonly combined with total tau protein as a ratio, can identify AD pathology long before dementia by distinguishing CH people with normal versus abnormal CSF biomarkers of AD: CH participants with normal Aβ_42_/Tau (CH-NAT) versus CH participants with abnormal or pathological Aβ_42_/Tau (CH-PAT; Fagan et al., 2007).

Here, we examined if lipid metabolism differed in CH volunteers negative or positive for AD biomarkers, or those with clinical AD, by measuring GP and SP lipid classes and their molecular species in CSF supernatant fluids (SF) and membrane nanoparticles (NP). We tested the hypothesis that lipolysis contributes to the pathological process by measuring the activity of two enzymes that hydrolyze membrane lipids: phospholipase A_2_ (PLA_2_) and sphingomyelinase (SMase) activities. PLA_2_ activity has been shown to increase in CSF in AD and associates with amyloid plaques (Stephenson et al., 1996), while SMase activity changes in CSF and brain fractions from AD patients (Jana and Pahan, 2004; Lee et al., 2014; Fonteh et al., 2015). Our data show an accumulation of GP in the supernatant fluid and SPs in the membrane nanoparticles of CH study participants with CSF AD biomarkers and lower lipid compositions in samples from those with clinical AD. We propose that our findings of enhanced lipid turnover in CH individuals with AD biomarkers, followed by lipolysis in the AD dementia stage, may be useful for targeting and testing AD treatments.

## Materials and Methods

### Clinical Methods

The local Institutional Review Board of Huntington Memorial Hospital approved our protocol and consent form, and study participants gave written informed consent. Clinical details of our classification have been reported (Harrington et al., 2013). In brief, 70 participants were classified at a clinical consensus conference using UDS and NACC criteria (Harrington et al., 2013) as CH based on their having no evidence of cognitive impairment after uniform clinical and neuropsychological examinations. Of 40 participants with dementia, 29 were diagnosed after fulfilling the criteria for clinically probable AD (Harrington et al., 2013). Participants completed the National Cancer Institute Diet History Questionnaire (DHQ; Subar et al., 2001, Version 2, 2010) that estimates the annual intake of 176 nutrients and food groups. We only trusted the memories of the CH subgroups for reliable DHQ data. Answers on paper questionnaires were entered online by study staff, and the Diet^∗^Calc 1.5 software was used to obtain dietary intake measurements. Fasting CSF and blood samples were obtained between 8:00 and 10:00 h within one month of neuropsychological testing, prepared and stored within 2 h of collection in polypropylene tube aliquots. Lumbar CSF was immediately examined for cells and total protein, and the remainder stored in 1 mL aliquots at −80∘C.

### CSF Aβ_42_ and Tau

The concentrations of CSF Aβ_42_ and Tau were measured using a sandwich enzyme-linked immunosorbent assay kit ([ELISA] Innotest β-amyloid_(1–42)_ and Innotest hTAU-Ag, Innogenetics, Gent, Belgium) according to the manufacturer’s protocol (Harrington et al., 2013). In brief, to determine the concentration of Aβ_42_, we added 25 μL of CSF sample and standards in duplicate into the monoclonal antibody (21F12) pre-coated plate and incubated with biotinylated antibody (3D6). We determined the Aβ_42_ concentration using a standard curve, between 125 and 2,000 pg/mL, with an assay detection limit of ± 50 pg/mL. In the Tau ELISA assay, we added 25 μL of CSF sample and standards in duplicate into the monoclonal antibody (AT120) pre-coated plate and incubated overnight with two biotinylated Tau-specific antibodies (HT7 and BT2). We quantified tau in CSF samples using a standard curve ranging between 75 and 1,200 pg/mL and an assay detection limit of ± 59.3 pg/mL.

### Determination of Protein in CSF

Supernatant fluids and NP fractions were diluted using phosphate-buffered saline, and protein contents were determined using a fluorescence-based Quant-iT^TM^ Protein Assay detection kit (Invitrogen, Eugene, OR, united States) with 0–500 ng BSA as standards.

### Nanoparticle (NP) Quantification in CSF

We determined the number and size distribution of NPs in CSF using a NanoSight NS300 instrument (Malvern Panalytical, Inc., Westborough, MA, United States; Fonteh et al., 2020). Briefly, after centrifugation at 3,000 RCF for 3 min to remove cellular debris, we diluted CSF (10×) using dd-H_2_O. The diluted CSF was continuously infused into the NS300 previously calibrated with polystyrene beads (30, 100, and 400 nm). Light scattering data was recorded at 432 nm for 60 s (×5) and processed using the Nanoparticle Tracking Analysis software (Malvern Panalytical, Inc.).

### CSF Fractionation and Glycerophospholipid Extraction

Supernatant fluids and NP fractions were isolated from CSF as described (Harrington et al., 2009). Briefly, starting with 4 mL of CSF (SF1), we obtained SF2 and NP2 after centrifugation at 17,000 *g*. SF2 was centrifuged again at 200,000 *g* to obtain the SF and NP fractions. The SF fraction was stored at −80°C, and the NP containing CSF nanoparticles was washed with 4 mL of phosphate-buffered saline, re-pelleted at 200,000 g, and re-suspended in 50 μL of phosphate-buffered saline.

### Materials for Lipid Analyses

HPLC grade water, 2-isopropanol, and acetonitrile (ACN) were purchased from VWR (West Chester, PA, United States). Ammonium acetate and butylated hydroxyl toluene (BHT) were purchased from Sigma (St Louis, MO, United States). Phosphatidylcholine (17:0 PC and 11:0 PC), phosphatidylethanolamine (17:0 PE), phosphatidylserine (17:0 PS) and lysophosphatidylcholine (11:0 LPC), sphingomyelin (SM), ceramide (CM), and dihydroceramide (dhCM) standards were purchased from Avanti Polar Lipids (Alabaster, AL). N-arachidonoyl phosphatidylethanolamine (NAPE), D_4_-platelet-activating factor (PAF), and D_4_-lysophosphatidylcholine (LPC) were purchased from Cayman Chemical (Ann Arbor, MI, United States).

### Lipid Extraction

Internal standards (IS) and retention time calibrants [11:0 PC (5 ng), D_4_-PAF (1 ng), 11:0 LPC (5 ng), and D_4_-LPC (5 ng)] were added to 1 mL SF from the original 4 mL of CSF, and GPs were extracted using a modified Bligh and Dyer (1959) procedure. Briefly, to limit lipid oxidation, we added 2 mL methanol containing 0.2 mg/mL BHT and performed all extraction at room temperature. A lipid-rich chloroform layer was aspirated to clean borosilicate culture tubes. Similarly, 40% of NP from the original 4 mL of CSF was suspended in 1 ml water containing 1 M NaCl and extracted as described above. The GP-rich chloroform layers from SF or NP were dried under a stream of N_2_ and reconstituted in HPLC solvent for LC/MS analyses.

### Hydrophilic Interaction Liquid Chromatography (HILIC) of GPs and SPs

Hydrophilic interaction liquid chromatography was performed using an HP-1100 system equipped with an autosampler, a column oven maintained at 35∘C, and a binary pump system using TSK-Gel Amide-80 Column (2.0 × 150 mm). GPs and SPs were isolated using a binary solvent system of 20% acetonitrile in isopropanol (Solvent A) containing 8% solvent B (20% water in isopropanol containing 10 mM ammonium acetate) at a flow rate of 0.2 ml/min (Fonteh et al., 2013, 2015). Solvent A was maintained for 5 min followed by a linear increase to 20% solvent B in 20 min, maintained at 20% B for 25 min, and then equilibrated with solvent A for 15 minutes before subsequent injections.

### Positive Ion Electrospray Ionization (ESI) Mass Spectrometry (MS)

Glycerophospholipids and SPs eluting from the HILIC column were positively ionized using an electrospray ionization (ESI) probe and detected using several MS scanning modes in a TSQ mass spectrometer (Thermo Fisher Scientific, San Jose, CA, United States; Fonteh et al., 2013, 2015). The MS was operated with a spray voltage of 4.5 kV, a heated capillary temperature of 300∘C, and nitrogen (50 units) and argon (5 units) as the sheath gas and the auxiliary gas, respectively. All GPs (PE, PE1, NAPE, PC, C11:0 PC internal standard, PS, PAF-like lipids [PAF-LL], and LPC) and SPs (SM, CM, dhCM) scans were optimized for collision energies, acquisition mass range, and retention times. To better isolate lipid classes, we collected data using three different scan windows from 0–5, 5–16, and 16–40 min (Fonteh et al., 2013, 2015).

### GP and SP Analyses

Peak areas for all GPs were integrated using the Qual Browser module of the Xcalibur software (Thermo Fisher, San Jose, CA, United States) normalized to the IS, C11:0 PC. For quantification, standard curves of GPs and SPs were obtained from each lipid amount plotted against each lipid’s intensity ratio to the C11:0-PC internal standard. Quantities of GPs and SPs were calculated for SF (ng/ml CSF) or NP (ng/ml CSF equivalent) using the ensuing standard curves. PC, PE, PS, and SM molecular species were identified using the Qual browser’s spectra function. The major peaks were identified using Lipid Maps MS tools^1^.

### Phospholipase A_2_ (PLA_2_) Activity Assay

A modified liposomal-based fluorescent assay was used to measure PLA_2_ activity in CSF samples. Briefly, a PLA_2_ substrate cocktail consisting of 7-hydroxycoumarinyl-arachidonate (0.3 mM), 7-hydroxycoumarinyl-linolenate (0.3 mM), hydroxycoumarinyl-6-heptanoate (0.3 mM), 10 mM dioleoylphosphatidylcholine (DOPC), and 10 mM dioleoylphosphatidylglycerol (DOPG) was prepared in ethanol. Liposomes were formed by gradually adding 77 μl substrate/lipid cocktail to 10 ml PLA_2_ buffer [50 mM Tris-HCl (pH 8.9), 100 mM NaCl, 1 mM CalCl_2_] while stirring rapidly over 1 min using a magnetic stirrer. CSF containing 10 μg total protein was added to 96 well plates, and PLA_2_ activity was initiated by adding a 50 μl substrate cocktail. Fluorescence (Excitation at 360 nm and emission at 460 nm) was measured, and specific activity was determined [relative fluorescent units (RFU)/μg protein] for each sample determined (Fonteh et al., 2013).

### Sphingomyelinase (SMase) Activity Assay

Fluorescence (Excitation at 360 nm and emission at 460 nm) was measured, and specific activity was determined (RFU/μg protein) for each sample (Fonteh et al., 2015).

### Statistical Analyses

All GP and SP data are presented as the mean ± SEM with 95% CI for each cognitive or Aβ_42_/Tau subgroups. One way ANOVA on ranks (Kruskal–Wallis test) and correction for multiple comparisons using statistical hypothesis testing using Dunn’s method were performed to determine within-group differences of GPs and SPs classes and molecular species. Spearman correlation was used to determine the association of lipids species. One way ANOVA analyses were performed using GraphPad Prism software (La Jolla, CA, United States). Data normalization was performed using MetaboAnalyst software. Briefly, GP and SP data were converted to tab-delimited text (.txt) before import into the MetaboAnalyst Statistical Analysis platform (Mendes et al., 2018; Chong and Xia, 2020). Data normalization and scaling used globalized logarithm transformation (glog) and mean-centering to obtain a Gaussian distribution and compare lipid levels over several orders of magnitude in the SF and NP fractions. Hierarchical clustering data presented in the form of a heatmap used Euclidean for distance measure and Ward for the clustering algorithm (Mendes et al., 2018; Chong and Xia, 2020). Data were considered significant if *p* < 0.05 after adjustment for multiple comparisons.

## Results

### CSF Aβ_42_ and Tau Levels Establish the Presence of AD Biomarkers in Some CH Individuals

An expert research consortium offered a working definition of three preclinical stages of AD pathology, based on molecular and neuroimaging biomarkers (Mendes et al., 2018): stage 1, CH with abnormal amyloid in CSF or brain; stage 2 adds to evidence of neurodegeneration to stage 1; stage 3 adds subtle cognitive change (insufficient to diagnose MCI) to stage 2. The authors modeled these preclinical stages to hypothetical pathophysiology that merits further dissection to unravel the diversity of dementia risk factors and the heterogeneous pathophysiology, exemplified by cases confirmed with AD at autopsy, but with prior normal amyloid imaging or normal CSF Aβ_42_. To study CH individuals with early AD pathology and compare it with dementia, we analyzed CSF Aβ_42_ and tau from individuals with clinical diagnoses ranging from healthy to dementia, including a CH cohort with an increased likelihood of preclinical pathology because of advancing age (Harrington et al., 2013). Our study participants with normal cognitive function are separated into two subgroups based on CSF Aβ_42_/Tau levels that have either CH-NAT or CH-PAT, Table 1. The CH-PAT group is cognitively healthy based on normal neuropsychometry, but with significantly lower Stroop Color Word performance than the CH-NAT group (Harrington et al., 2013), suggesting early executive function deterioration. Longitudinal follow-up over 4 years shows that 40% of CH-PAT participants cognitively decline while none of the CH-NAT participants decline (Harrington et al., 2019). Our CH subgroup with normal CSF protein, CH-NAT, fits Stage 0, while the subgroup with abnormal protein (CH-PAT) includes stages 1 and 2.

**TABLE 1:** Demographic data, mini-mental state examination scores (MMSE), and Aβ_42_/tau of cognitively healthy (CH) and Alzheimer’s disease (AD) participants.

Parameters	CH-NAT (*n* = 35)	CH-PAT (*n* = 33)	AD (*n* = 25)	*P*-value^#2^
Age, mean, SD (95% CI)	77 ± 7 (74-79)	78 ± 7 (75-80)	75 ± 9 (72-79)	0.6876
Female (n female/n total)^#1^	25/35	20/33	12/25	
**Neuropsychology data (mean, SD, 95% CI)**				
MMSE	28.9 ± 1.2	28.5 ± 1.8	15.9 ± 7.8	< 0.0001
**Amyloid and tau (means SD, 95% CI)**				
Aβ_42_ (pg/ml)	898 ± 216 (825-971)	487 ± 213 (410-566)	458 ± 199 (373-542)	< 0.0001
tau (pg/ml)	216 ± 121 (175-257)	345 ± 187 (276-413)	505 ± 200 (421-590)	< 0.0001
Aβ_42_/T-tau	4.9 ± 1.8 (4.2-5.5)	1.7 ± 0.8 (1.4-1.9)	1.0 ± 0.5 (0.8-1.2)	< 0.0001

*^#1^Fisher‘s Exact test showed no significant difference in the female to male ratio for CH-NAT and CH-PAT (p (=0.3458), CH-NAT, and AD (p (=0.0.0657), and CH-PAT and AD (p (=0.3391). ^#2^One way ANOVA (p-values) and Dunn’s multiple comparison test was performed for MMSE, and Aβ_42_, tau, and Aβ_42/_tau ratio. MMSE scores are similar in the CH groups but significantly higher than the AD group. Aβ_42_ levels are higher in the CH-NAT group than in CH-PAT and AD. Tau is lower in CH-NAT compared with CH-PAT and AD resulting in a significantly higher Aβ_42_/tau ratio in CH-NAT than in AD.*

### GP Metabolism Is Increased in CH Individuals With AD Biomarkers

We investigated whether GP classes (PC, LPC, PAF_LL, PE, and PS; Fonteh et al., 2013) and their molecular species differed between the CH-NAT, CH-PAT, or the AD groups.

### PC Changes in the SF and NP Fractions

**FIGURE 1:**
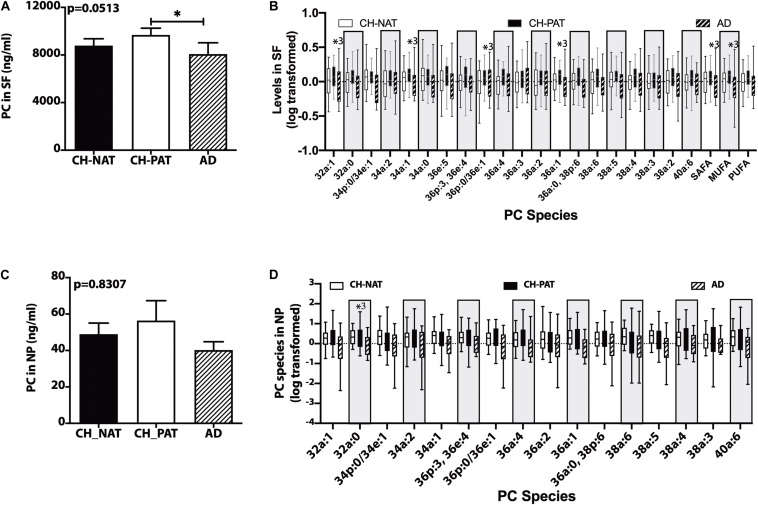
Phosphatidylcholine (PC) and PC species in the supernatant fluids (SF) and nanoparticles (NP) fractions – Levels of PC in the SF fraction **(A)** and PC species in the SF fraction **(B)**, PC in the NP fraction **(C)**, and PC species **(D)** in the NP fractions were determined using Hydrophilic interaction liquid chromatography (HILIC) chromatography coupled with mass spectrometry. The mean ± SEM for PC and PC species were normalized and log 10 transformed to display species of different concentrations. Group comparisons were made using One way ANOVA (Kruskal–Wallis test) combined with Dunn’s multiple comparisons. The *p*-values are from One way ANOVA, and significant changes (*p* < 0.05) from Dunn’s are indicated with an asterisk(*).

In the SF fraction, there is a significant increase in PC in CH-PAT compared to AD (Figure 1A). Further examination showed five PC species, PC-SAFA, and PC-MUFA exhibit group differences (Figure 1B). Multiple comparisons show significantly higher levels of these PC species in CH-PAT compared with AD. Similar to the SF fraction, there is a trend for higher PC levels in the NP fraction of CH-PAT compared with CH-NAT and AD (Figure 1C). The examination of PC species shows a higher PC32a:1 in CH-PAT than in AD (Figure 1D).

### Other Choline-Containing GPs in the SF and NP Fractions

**FIGURE 2:**
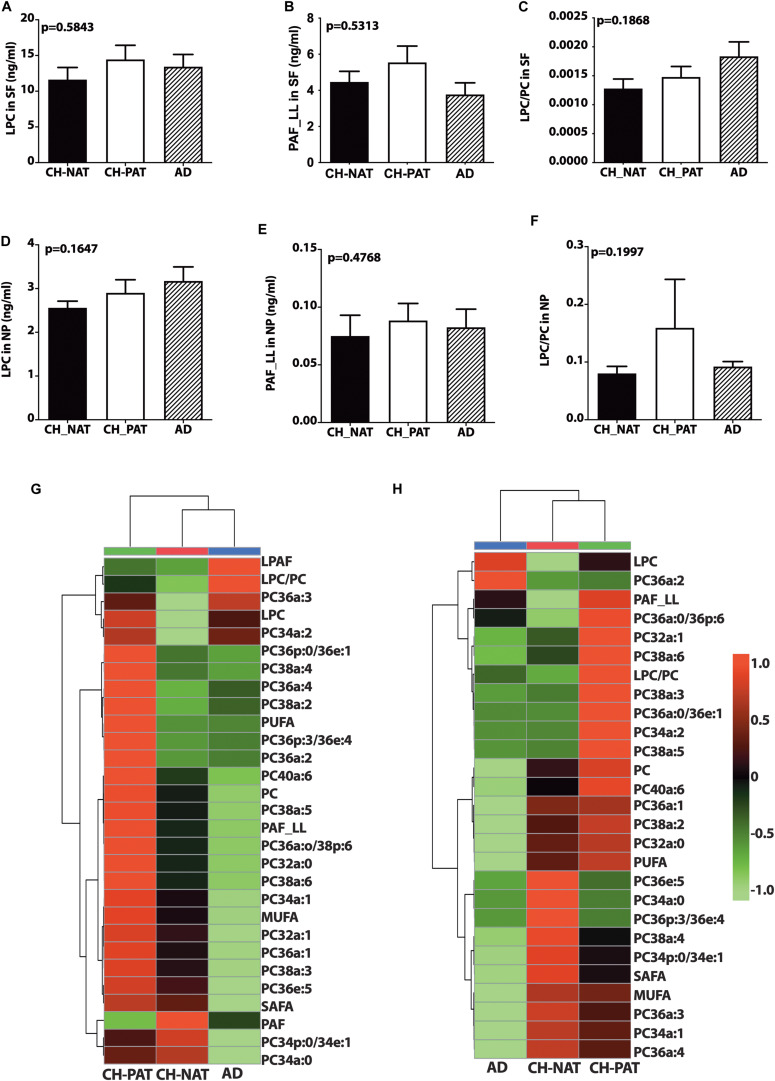
Phosphocholine glycerophospholipids and hierarchical clustering of all phosphatidylcholine (PC) glycerophospholipids in the supernatant fluids (SF) and nanoparticles (NP) fractions – Levels of lysophosphatidylcholine (LPC; **A**), PAF_LL **(B)**, and the LPC to PC **(C)** ratios were determined in the SF fraction of CH-NAT, CH-PAT, and AD. LPC **(D)**, and PAF_LL **(E)** and the LPC/PC ratio **(F)** were similarly determined in the NP fractions. Group comparisons were made using One way ANOVA (Kruskal–Wallis test) combined with Dunn’s multiple comparisons. The *p*-values are from One way ANOVA, while significant changes (*p* < 0.05) from Dunn’s are indicated with an asterisk (*). The heatmap displays the hierarchical clustering of PC-glycerophospholipids in the SF fraction **(G)** and the NP fraction **(H)**. Distance measures of the heatmap use Euclidean while the clustering algorithm uses Ward on the Metabanalyst 4.0 platform.

There is a trend for higher LPC (Figure 2A) and PAF_LL (Figure 2B) in CH-PAT and a progressive increase in the LPC/PC ratio in CH-PAT and AD (Figure 2C) in the SF fraction. We observe a similar increase in LPC (Figure 2D), PAF-LL (Figure 2E), and the LPC/PC (Figure 2F) ratio in the NP fraction of CH-PAT.

Figures 2G,H are the clustering result illustrated as a heatmap showing changes in the PC measures in the SF and NP fractions, respectively. In general, the mean levels of GPs are higher in the CH-PAT than the CH-NAT group, which contrasts with the general decrease of GPs in AD. Of the 30 choline-containing GP measures in the SF fraction, three are highest in CH-NAT, three measures are highest in AD, while the rest of the measures are highest in CH-PAT (Figure 2G). In the NP fraction, two choline-containing GP measures are highest in AD, 10 measures are highest in CH-NAT, and 15 are highest in CH-PAT (Figure 2H).

### Choline-Containing GPs Normalized to the Number of NPs

**FIGURE 3:**
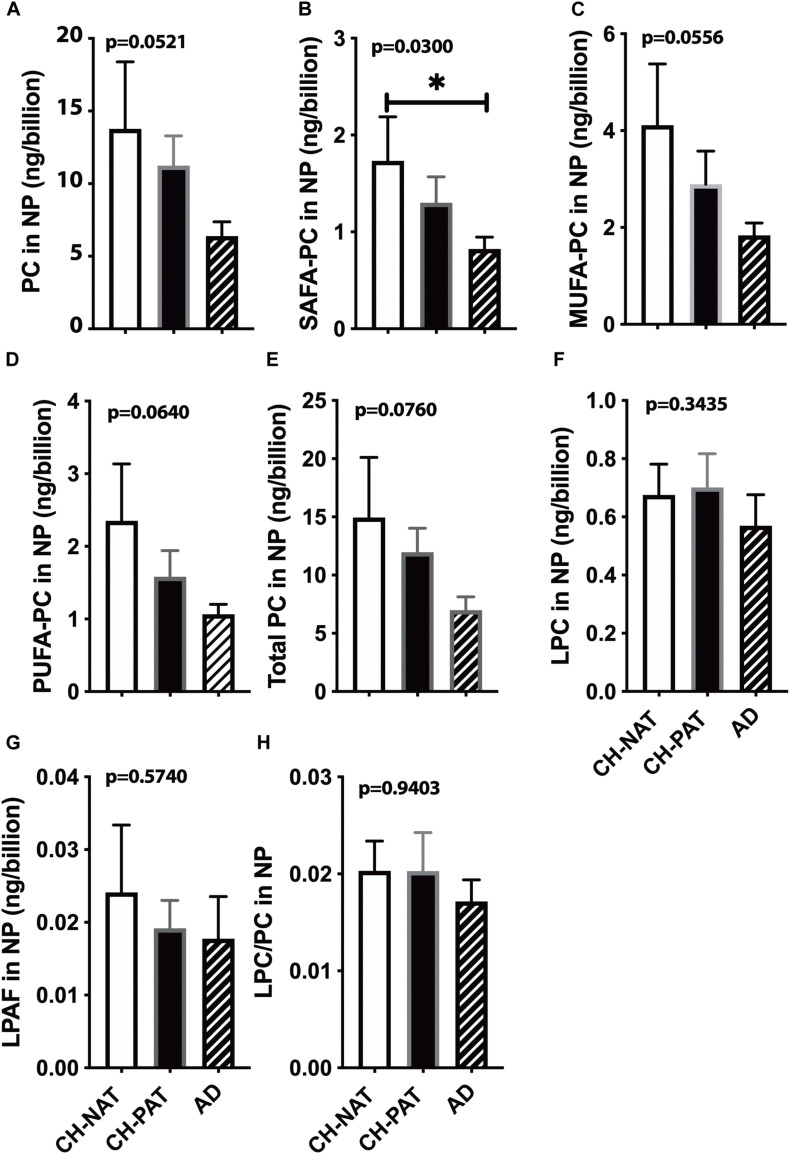
Phosphatidylcholine (PC) levels normalized to the nanoparticles (NP) concentration in cerebrospinal fluid (CSF) – The levels of PC **(A)**, SAFA-containing PC **(B)**, MUFA-containing PC species **(C)**, PUFA-containing PC species **(D)**, LPC **(E)**, LPAF **(F)**, total PC lipids **(G)**, and the LPC to PC ratio **(H)** were normalized to the number of NPs (billions) in CSF. These data are the mean ± SEM, and group comparisons were made using One way ANOVA (Kruskal–Wallis test) combined with Dunn’s multiple comparisons. The *p*-values are derived from One way ANOVA, and significant changes (*p* < 0.05) from Dunn’s are indicated with an asterisk (*).

We normalized NP lipids levels to the number of particles to determine if the membrane concentration could account for group differences. When normalized to the number of NPs in CSF, we find a progressive decrease in PC (Figure 3A), SAFA-PC (Figure 3B), MUFA-PC (Figure 3C), PUFA-PC (Figure 3D), and total choline-containing GPs (Figure 3E). We do not observe similar changes in LPC (Figure 3F), LPAF (Figure 3G), and the LPC/PC (Figure 3H) ratio in the NP fractions.

### Correlation of PC to LPC

**FIGURE 4:**
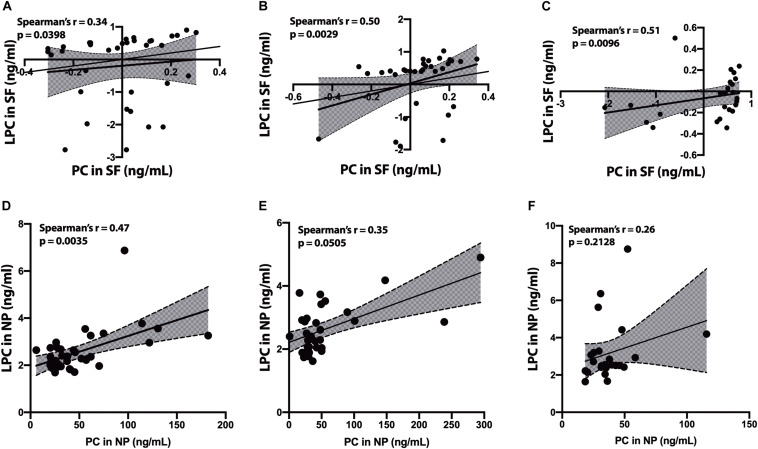
Correlation of PC to lysophosphatidylcholine (LPC) in cerebrospinal fluid (CSF) fractions – We performed Spearman’s correlation of PC to LPC in the SF fraction for CH-NAT **(A)**, CH-PAT **(B)**, and AD **(C)**. Similarly, we performed a correlation of PC to LPC in the nanoparticles (NP) fraction for CH-NAT **(D)**, CH-PAT **(E)**, and AD **(F)**. The data for the SF fractions was log 10 transformed to enable us to plot all the data on the same scale while the unnormalized data is shown for the NP correlation analyses. Spearman’s rho (*r*) and the *p*-values are indicated for each correlation analysis.

In the SF fraction, there is positive correlation between PC and LPC in CH-NAT (Figure 4A, *r* = 0.34, *p* = 0.0398) and this level of correlation increases in CH-PAT (Figure 4B, *r* = 0.50, *p* = 0.0029), and in AD (Figure 4C, *r* = 0.51, *p* = 0.0096). In contrast to the SF fraction, PC correlates better in the NP fraction of CH-NAT (Figure 4D, *r* = 0.47, *p* = 0.0035) than in CH-PAT (Figure 4E, *r* = 0.35, *p* = 0.0505) and AD (Figure 4F, *r* = 0.26, *p* = 0.2128).

### Phosphatidylethanolamine (PE) and Phosphatidylserine (PS) in SF and NP Fraction

**FIGURE 5:**
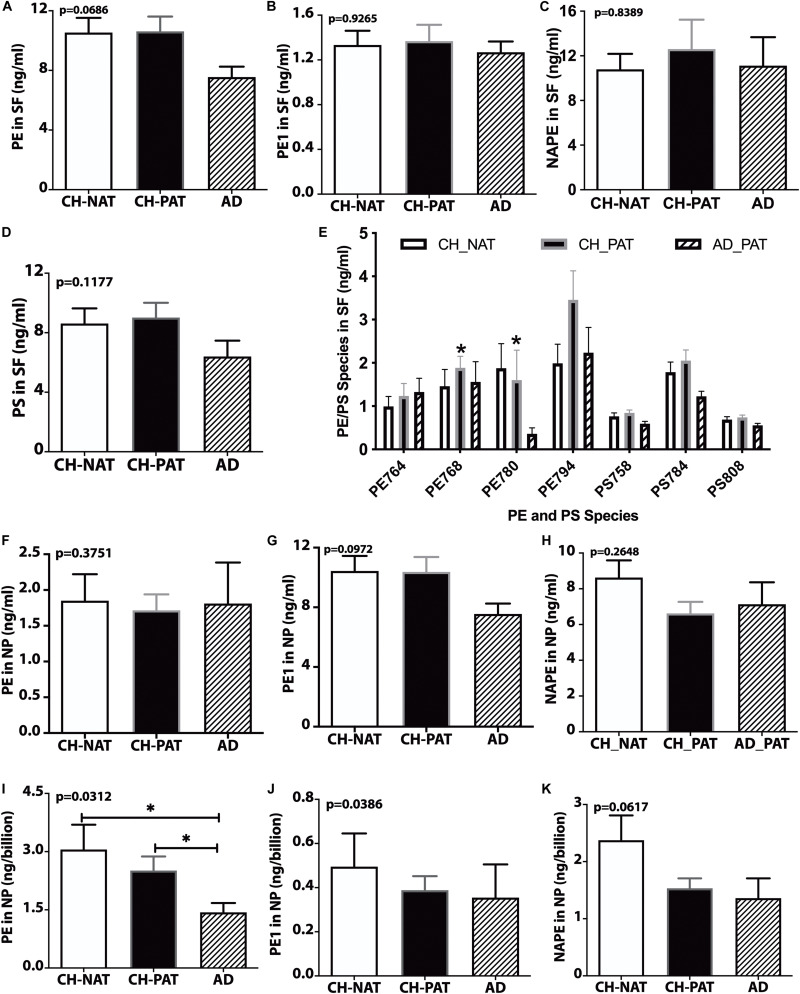
PE, PS, and their molecular species in cerebrospinal fluid (CSF) fractions – PE **(A)**, PE1 **(B)**, NAPE **(C)**, PS **(D)**, and PE and PS **(E)** species in the SF fractions are plotted for CH-NAT, CH-PAT, and AD. PE species identified in CSF include PE38a:8 (PE764), PE38a:5 (PE768), PE40p:4 (P780), and PE40a:5 (PE794). PS species include PS34a:3 (PS758), PS36a:4 (PS784), and PS38a:6 (PS808). **(F–H)** show the levels of PE, PE1, and NAPE in the nanoparticles (NP) fractions of CSF of CH-NAT, CH-PAT, and AD. For these same glycerophospholipids, we normalized the levels of PE **(I)**, PE1 **(J)**, and NAPE **(K)** to the number of nanoparticles. These data are the mean ± SEM and significant changes in GP amounts (*p* < 0.05) are indicated with a symbol (*). Group comparisons were made using one-way ANOVA (*p*-values) and Dunn’s multiple comparisons (asterisks).

We did not measure significant clinical group changes in PE (Figure 5A), PE1 (Figure 5B), NAPE (Figure 5C), and PS (Figure 5D) in the SF fractions. However, one PE species (PE38a:5) was significantly higher in CH-PAT than in CH-NAT, and a plasmalogen PE specie (PE40p:4) was significantly higher in CH-PAT than in AD (Figure 5E). In the NP fraction, we did not measure group differences in the levels of PE (Figure 5F), PE1 (Figure 5G), and NAPE (Figure 5H). However, when normalized to the number of NPs to determine if membrane concentration varied between groups, there was a significant decrease in PE in CH-PAT and AD compared with CH-NAT (Figure 5I), but not in the levels of PE1 (Figure 5J). Mean NAPE levels generally trended lower in CH-PAT and AD than in CH-NAT (Figure 5K).

### GP Summary

These data show subfraction-specific changes in GP classes and molecular species that reflect altered lipid metabolism from both extracellular fluid-derived SF and brain tissue-derived NP in the CH group with AD biomarkers. The increase of GPs in this CH-PAT group is most notable for being in the opposite direction from AD, in which the GPs other than LPC decrease.

### Changes in Sphingolipids (SPs) in Preclinical AD

Sphingolipids are essential in brain structure and function and are implicated in AD pathology (van Echten-Deckert and Walter, 2012; Martinez Martinez and Mielke, 2017). Therefore, we compared SP levels in our CH groups with normal or pathological Aβ_42_/tau ratio and with the AD group (Harrington et al., 2019). The SP classes we quantified in CSF fractions include SM, CM, and dhCM (Fonteh et al., 2015).

### SM Changes in the SF and NP Fractions

**FIGURE 6:**
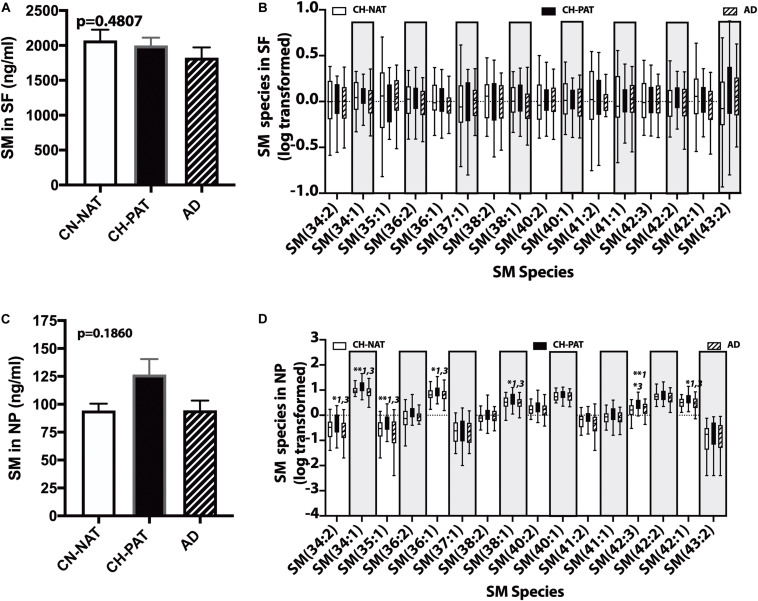
Changes in cerebrospinal fluid (CSF) sphingomyelin in the CH-PAT group – SM **(A)** and SM species levels **(B)** in the SF fractions were determined for CH-NAT, CH-PAT, and AD samples. We also determined SM **(C)** and SM species **(D)** levels in the nanoparticles (NP) fraction. The untransformed data are plotted for SM (mean ± SEM), while the log 10 transformed data (Tukeys plot) is presented for SM species. Multiple comparison tests (Dunns) significance is indicated with an asterisk and a number for CH-NAT versus CH-PAT (*1), CH-NAT, and AD (*2), and CH-PAT and AD (*3).

In the SF fraction, there is a trend for lower levels of SM in CH-PAT and AD compared to CH-NAT (Figure 6A). Examining SM species levels showed no significant differences between our three clinical groups (Figure 6B). In contrast to the SF fraction, SM levels trended on a higher side for CH-PAT than CH-NAT (Figure 6C), and seven SM species were significantly higher in CH-PAT than in CH-NAT and AD (Figure 6D).

### Other Sphingolipids in the SF and NP Fractions

**FIGURE 7:**
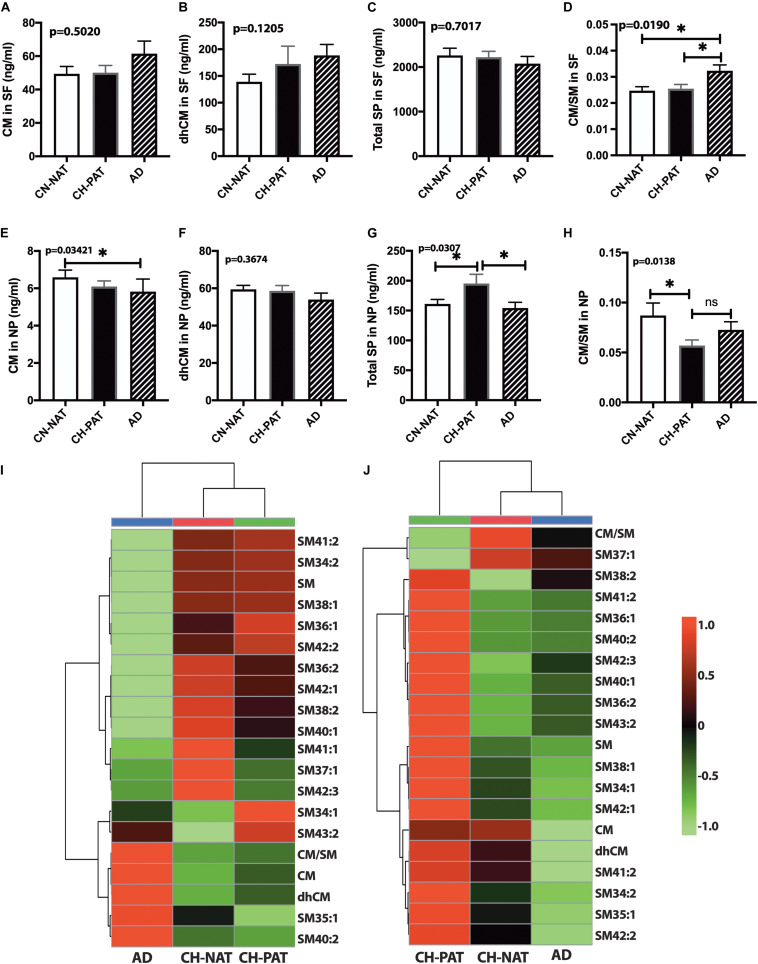
Alteration in cerebrospinal fluid (CSF) sphingolipids (SPs) in the CH-PAT group – SP levels in the supernatant fluids (SF) and nanoparticles (NP) fractions were determined for CH-NAT, CH-PAT, and AD samples. CM **(A)**, dhCM **(B)**, total SP in the SF fraction **(C)**, and the CM/SM ratio **(D)** were determined in the SF fraction. In the NP fraction, we also quantified CM **(E)**, dhCM **(F)**, total SP **(G)**, and the ratio of CM to SM **(H)**. These data are the mean ± SEM and a significant change (*p* < 0.05) in SP amounts way ANOVA and Dunns multiple comparisons) are indicated with a symbol (*). The heatmaps show the clustering data of SP changes in the SF fraction **(I)** and the NP fraction **(J)**. The distance measures used Euclidean while the clustering algorithm used Ward on the Metabanalyst 4.0 platform.

In the SF fraction, there is a trend for higher CM in AD (Figure 7A). While not significant, dhCM increases progressively in CH-PAT and AD compared with CH-NAT (Figure 7B), while total SP levels show a decreasing trend in CH-PAT and AD compared with CH-NAT (Figure 7C). The increase in CM concomitant with the SM decrease resulted in a significantly higher CM/SM ratio in AD than CH-NAT and CH-PAT in the SF fraction (Figure 7D). In the NP fraction, the mean CM level is significantly lower in AD than in CH-NAT (Figure 7E). In contrast to the increase of dhCM in the SF fraction (Figure 7B), dhCM levels trend lower in the NP fraction of AD (Figure 7F). Total SP in the NP fraction is significantly higher in CH-PAT than in CH-NAT and AD (Figure 7G). The NP fraction’s CM/SM ratio is significantly lower in CH-PAT than in CH-NAT (Figure 7H). Figures 7I,J are clustering results illustrated as a heatmap showing changes in 20 SP measures in the SF and NP fractions, respectively. Of the 20 SP measures in the SF fraction, seven are highest in CH-NAT, five are highest in AD, while the rest of the measures are highest in CH-PAT (Figure 7I). In the NP fraction, three SP measures are highest in CH-NAT, and 17 measures are highest in CH-PAT (Figure 7J).

### SM Levels Normalized to the Number of NPs

**FIGURE 8:**
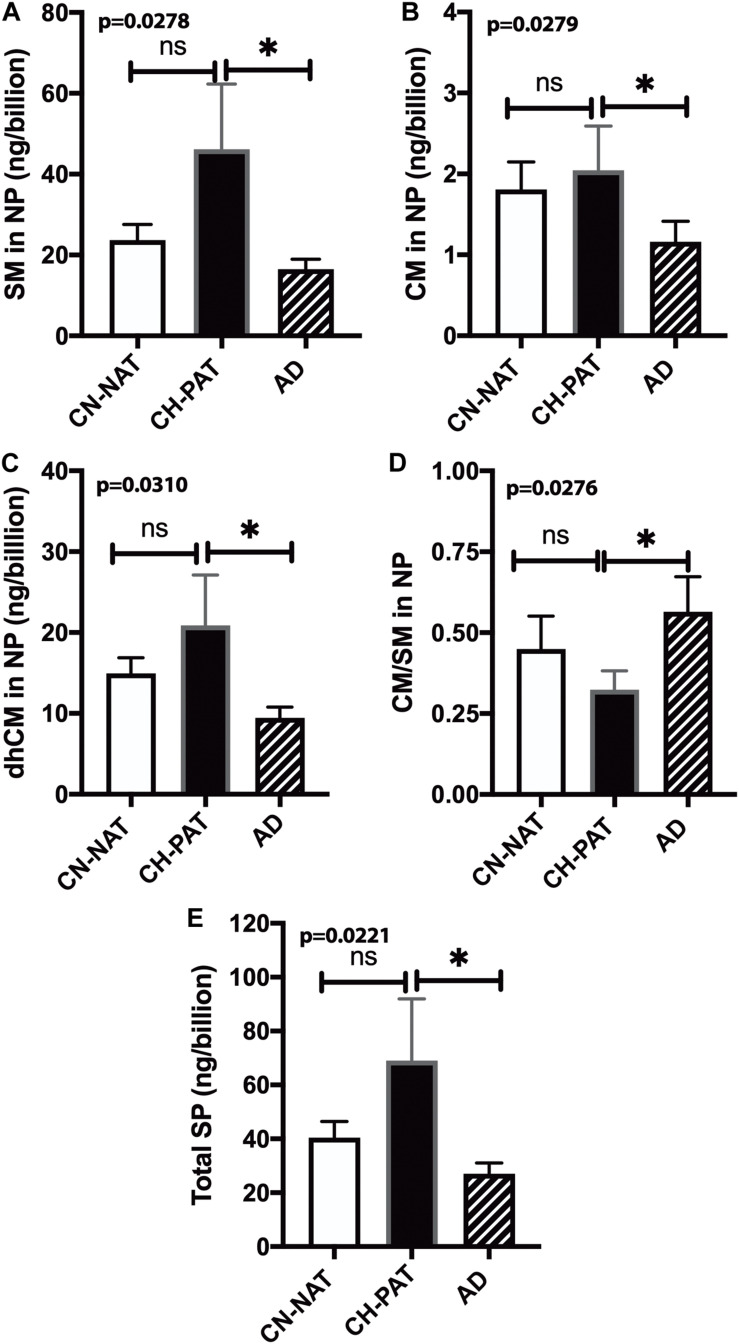
Sphingomyelin levels normalized to the nanoparticles (NP) concentration in cerebrospinal fluid (CSF) – The levels of SM **(A)**, CM **(B)**, dhCM **(C)**, CM/SM ratio **(D)**, and total SP **(E)** in the NP fraction were normalized to the number of NPs (billions) in CSF. These data are the mean ± SEM, and group comparisons were made using One way ANOVA (*p*-values) combined with Dunn’s multiple comparisons (asterisks). Significant changes (*p* < 0.05) are indicated with an asterisk (*).

To determine if membrane SP concentrations varied between groups, we normalized NP lipids levels to the number of particles. Normalized SM (Figure 8A), CM (Figure 8B), dhCM (Figure 8C) are significantly higher in CH-PAT than in AD. The SM and CM changes result in a significantly lower CM to SM ratio in CH-PAT than in AD (Figure 8D). Total SP per billion NPS is significantly higher in CH-PAT (Figure 8E).

### Correlation of SM to CM

**FIGURE 9:**
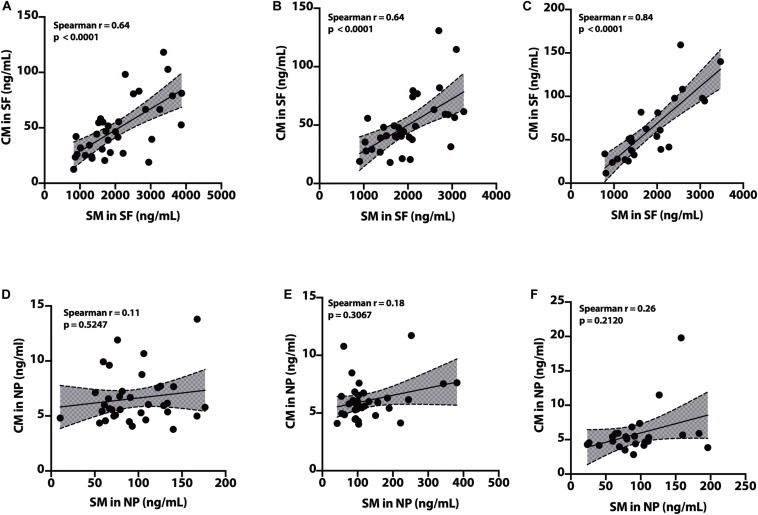
Correlation of SM to CM in cerebrospinal fluid (CSF) fractions – We performed Spearman’s correlation of SM to CM in the SF fraction for CH-NAT **(A)**, CH-PAT **(B)**, and AD **(C)**. Similarly, we performed a correlation of SM to CM in the nanoparticles (NP) fraction for CH-NAT **(D)**, CH-PAT **(E)**, and AD **(F)**. Spearman’s rho (*r*) and the *p*-values are indicated for each correlation analysis.

In the SF fraction, there is positive correlation between SM and CM in CH-NAT (Figure 9A, *r* = 0.64, *p* < 0.0001), CH-PAT (Figure 9B, *r* = 0.64, *p* < 0.0001), and in AD (Figure 9C, *r* = 0.84, *p* < 0.0001). In contrast to the SF fraction, SM does not correlates with CM in the NP fraction of CH-NAT (Figure 9D, *r* = 0.11, *p* = 0.5247), CH-PAT (Figure 9E, *r* = 0.18, *p* = 0.3067) and AD (Figure 9F, *r* = 0.26, *p* = 0.2120).

### Summary of SP Data

No changes in SM molecular species are measured in SF for the CH subgroups and AD. In contrast, there is an increase in 17 of 20 SP measures in CH-PAT compared with CH-NAT and AD. These data show an increase in membrane-bound SM and its main molecular species in CH-PAT, indicating modification in SP metabolism in the CH group with CSF biomarkers of AD. Modifications in SP metabolism may typify increased lipid membrane particle formation and enhanced oxidative stress in CH-PAT attributed to the increase in dhCM (Idkowiak-Baldys et al., 2010; Munoz-Guardiola et al., 2020) that may precede the neuronal apoptosis in AD. Thus, measuring SP in CSF fractions can be crucial in monitoring early AD pathology or metabolic screening modifiers at the earlier stages of AD pathology even before clinical features are evident.

### Increased PLA_2_ Activity Contributes to GP Lipolysis in AD Dementia

**FIGURE 10:**
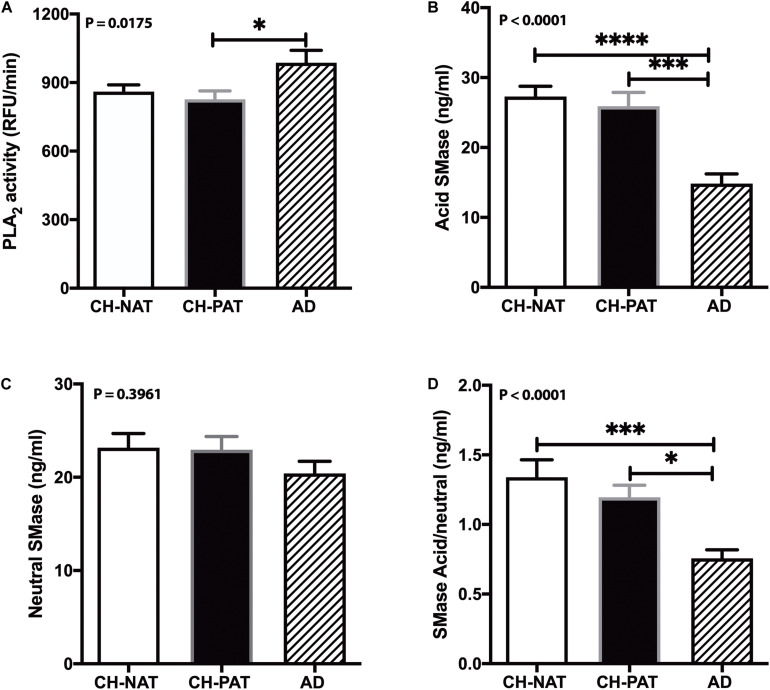
Phospholipase A_2_ (PLA_2)_ and SMase activity in cerebrospinal fluid (CSF) – PLA_2_ activity in CSF was determined for CH-NAT, CH-PAT, and AD **(A)**. Similarly, aSMase **(B)** and nSMase **(C)** activities were measured in CSF using a coupled fluorescent assay. The ratio of the acid SMase to nSMase activity is shown in **(D)**. These data are the mean ± SEM, and group comparisons were made using One way ANOVA (*p*-values) combined with Dunn’s multiple comparisons (asterisks). * indicate *p* < 0.05, ** *p* < 0.01, *** *p* < 0.005, **** *p* < 0.0001.

Phospholipase A_2_ hydrolyzes membrane lipids, and cerebrospinal PLA_2_ activity increases in AD (Fonteh et al., 2013). Immunohistochemical studies associate PLA_2_ also with AD plaques in human brain samples (Stephenson et al., 1996). Therefore, we measured PLA_2_ activity in CSF to determine if higher lipolysis may account for the decrease in GP levels in AD fractions compared with the CH samples. Mean PLA_2_ activity was similar in the CH-NAT and CH-PAT groups but significantly increased in AD (Figure 10A). These data suggest that PLA_2_ activity does not contribute to GP metabolism in the absence of clinical signs of AD. However, the higher LPC and lower PC levels found in CSF supernatants and pellet fractions from AD participants may result at least partly from increased PLA_2_ activity.

### Correlation of Phospholipase A_2_ Activity With Lysophopholipids

Lysophosphatidylcholine in NP did not correlate with PLA_2_ activity for CH (*r* = 0.24, *p* = 0.0685) and AD (*r* = −0.32, *p* = 0.2159). LPC did not correlate with PLA_2_ activity in the NP (*r* = −0.09, *p* = 0.6495) and SF (*r* = 0.15, *p* = 0.4442) fraction of CH-NAT participants. In contrast, LPC correlated with PLA_2_ activity in the SF (*r* = −0.35, *p* = 0.0495) and NP (*r* = 0.41, *p* = 0.0270) fractions of CH-PAT. We detected LPAF and PAF in the SF but not in the NP fraction. LPAF significantly correlated with PLA_2_ activity from CH (*r* = 0.34, *p* = 0.0049) but not AD (*r* = −0.13, *p* = 0.6007) participants. LPAF (*r* = 0.44, *p* = 0.0090) and PAF (*r* = 0.37, *p* = 0.0379) correlated with PLA_2_ activity in CH-NAT but not CH-PAT and AD.

### Sphingomyelinase Activity

Our studies have detected acid sphingomyelinase (aSMase) and neutral sphingomyelinase (nSMase) activities in CSF (Fonteh et al., 2015). Since the changes in SMase activities can influence SP metabolism and influence AD pathology (Jana and Pahan, 2010; Lee et al., 2014; Dinkins et al., 2016), we measured both aSMase and nSMase activities in CSF from CH clinical subgroups and AD. Compared with CH-NAT, aSMase activity decreased in CSF from CH-PAT (5.2%) and in AD (45.6%, *p* < 0.05, Figure 10B). nSMase activity was only slightly decreased in CH-PAT (−1.0%) and in AD (−12%, Figure 10C) compare with CH-NAT. The ratio of aSMase to nSMase was significantly higher in CH-NAT and CH-PAT than in AD (Figure 10D). Although we detected acid and neutral SMase in CSF and their putative product, CM, neither activity correlated with SP levels in the SF and NP fractions (data not shown).

### Dietary Lipid Sources, Lipid Modulating Medications, and Lipid Transport in the Blood Do Not Explain the Lipid Changes in the Healthy, AD Biomarker Stage, or Dementia Stages

We surmise that the distinct changes in the SF and NP fractions’ lipid composition between our clinical groups represent brain pathology differences. However, altered lipids in the CSF fractions might originate from their local source, from altered clearance or delivery from the systemic circulation, different diets, or lipid modulating medications. To investigate these possibilities, we measured the mean fasting blood levels of triglycerides, cholesterol, HDL, and LDL, assessed diets over the previous year, and compared the prescription, over-the-counter, and nutritional supplements taken by the participants. Neither medications nor measured blood lipids differ between the CH-NAT, CH-PAT, and AD groups (Harrington et al., 2013; Chew et al., 2020). The comprehensive dietary questionnaire was only analyzed with confidence in the CH sub-groups to avoid any bias from impaired memories in those with dementia; the dietary lipid intake did not differ between CH-NAT and CH-NAT participants (Fonteh et al., 2020). These results suggest that the changes mentioned above in GPs and SPs in biochemical and dementia stages likely result from the brain pathophysiology.

## Discussion

The structural and functional importance of lipids underscores their roles in several brain disorders, yet brain lipid composition and turnover in early AD pathology has not received much study. Moreover, despite substantial progress in defining genetic, clinical, cellular, and molecular components of the pathophysiology (Laske and Stellos, 2014; Kumar et al., 2015; Koch et al., 2018; Guo et al., 2020), our knowledge of how the different dementia risk factors associated with early AD pathology is also insufficient for diagnosis and effective treatments. These two shortcomings are connected because of the substantial loss of the lipid-rich brain tissue in AD starts many years before symptoms arise. We hypothesize that changes in lipid metabolism are an important component of the biomarker/non-clinical AD stage. To test this, we developed methods to study the composition of brain-derived lipids in the CSF to assess *in vivo* brain metabolism. 30–200 nm-sized particles that we described in CSF to have neurotransmitters, lipids, active enzymes, and signaling functions and are likely derived from brain cells via exocytosis and vesicle signaling (Harrington et al., 2009). We also reported that these nanoparticles are rich in the GP class of lipids, with compartmentalization between the SF and NP fractions of CSF (Fonteh et al., 2013, 2015, 2020). Here, we provide evidence to support this hypothesis by finding that GPs and SPs in both SF and NP fractions are higher in CH-PATs and lower in dementia. In the SF fraction, the most significant changes are in the PC species in the SF fraction (Table 2). In the NP fractions, most of the changes occur with SM species Table 2). Levels of two major lipid metabolizing enzymes are also altered in AD (Table 2).

**TABLE 2:** Lipids and enzyme activities that change in cerebrospinal fluid (CSF) fractions from participants with normal Aβ_42_/tau compared with pathological Aβ_42_/tau and Alzheimer’s disease (AD) participants.

Lipids in SF fraction			
**Lipids**	**CH-NAT (*n* = 35)**	**CH-PAT (*n* = 33)**	**AD (*n* = 25)**
PC^#3^	8787 ± 3433 (7607.7-9966.2)*^a^*	9688.8 ± 3254.4 (8534.9-10842.8)	8076.4 ± 4746.4 (6117.1-10036.6)
PC-SAFA^#3^	931.8 ± 452.6 (778.7-1085)	945.5 ± 345.1 (823.1-1068)	760.9 ± 452.0 (574.3-947.4)
PC-MUFA^#3^	2600.4 ± 1067.7 (2239.1-2961.7)	2915.6 ± 1007 (2558.5-3272.6)	2279.6 ± 1372.3 (1713.2-2846.1)
PC32a:0	382.2 ± 148.8 (331.0-433.3)	439.6 ± 173.0 (378.3-500.9)	340.8 ± 201.0 (257.8-423.8)
PC34a:1^#3^	2247.6 ± 887.1 (1943-2552)	2503.6 ± 873.1 (2194.1-2813.2)	1954.4 ± 1207.2 (1447.1-2443.7)
PC36a:1^#3^	274.6 ± 104.9 (238.6-310.6)	300.3 ± 100.7 (264.6-336.0)	239.9 ± 135.8 (183.9-296.0)
PC36a:0^#3^	53.4 ± 21.2 (46.1-60.7)	59.7 ± 25.3 (50.8-68.7)	48.8 ± 36.7 (33.6-63.9)
PC34p:0/34e:1	95.7 ± 49.5 (78.7-112.7)	88.3 ± 35.4 (75.7-100.8)	71.3 ± 48.0 (51.5-91.1)
CM/SM^#2,#3^	0.025 ± 0.001 (0.02-0.03)	0.026 ± 0.01 (0.02-0.03)	0.032 ± 0.011 (0.028-0.037)
**Lipids in the NP fraction**			
	**CH-NAT (*n* = 35)*^*b*^***	**CH-PAT (*n* = 34)**	**AD (*n* = 25)**
PC32a:0	1.77 ± 1.07 (1.32-2.21)	3.0 ± 3.47 (1.75-4.35)	2.73 ± 2.69 (1.83-3.63)
CM^#2^	6.59 ± 2.29 (5.81-7.38)	6.10 ± 1.72 (5.49-6.71)	5.83 ± 3.35 (4.44-7.21)
Total SP^#1,#3^	161.1 ± 44.0 (132.2-184.9)	195.2 ± 87.8 (164.1-226.1)	154.3 ± 48.0 (134.5-174.2)
SM(34:2)^#3^	0.43 ± 0.35 (0.31-0.66)	0.71 ± 0.65 (0.45-0.93)	0.37 ± 0.43 (0.20-0.55)
SM(34:1)^#3^	10.83 ± 5.77 (8.85-12.81)	15.58 ± 10.31 (11.99-19.18)	9.63 ± 6.63 (6.89-12.36)
SM(35:1)^#1,#3^	0.44 ± 0.40 (0.30-0.57)	0.81 ± 0.67 (0.57-1.05)	0.39 ± 0.47 (0.19-0.59)
SM(36:2)^#1^	1.02 ± 0.82 (0.74-1.30)	1.68 ± 1.48 (1.16-2.19)	1.16 ± 1.10 (0.70-1.61)
SM(42:3^)#1^	1.77 ± 0.96 (1.44-2.10)	2.97 ± 2.04 (2.26-3.68)	2.16 ± 1.21 (1.66-2.66)
SM(42:1)	3.89 ± 2.30 (2.89-4.48)	4.88 ± 2.63 (3.96-5.80)	3.43 ± 2,04 (2.59-4.27)
CM/SM^#1^	0.09 ± 0.01 (0.06-0.11)	0.06 ± 0.03 (0.05-0.07)	0.07 ± 0.04 (0.06-0.09)
**Enzyme activities**			
	**CH-NAT (*n* = 36)*^*b*^***	**CH-PAT (*n* = 33)**	**AD (*n* = 23)**
PLA_2_^#3^	860.5 ± 173.1 (800.1-920.9)	827.1 ± 202.2 (752.9-901.2)	986.7 ± 230.9 (871.9-1102)
aSMase^#2,#3^	27.2 ± 8.7 (24.4-30.2)	25.9 ± 11.4 (21.9-29.9)	14.8 ± 6.7 (11.9-17.7)
aSMase/nSMase^#2,#3^	1.3 ± 0.8 (1.0-1.4)	1.2 ± 0.5 (1.02-1.37)	0.8 ± 0.3 (0.6-0.9)

*^a^Lipid levels and enzyme activities are presented as the mean ± SD and 95% confidence intervals of the mean. We compared lipid levels or enzyme activities in CSF fractions from CH-NAT, CH-PAT, and AD using One way ANOVA (Kruskal–Wallis test) with multiple comparisons (Dunn’s multiple comparisons). The above lipids and enzyme activities showed significant (p < 0.05) group differences. ^#1^ Indicates CH-NAT is different from CH-PAT, ^#2^ indicates CH-NAT is different from AD, and ^#3^ indicates CH-PAT is significantly different from AD using multiple comparison tests. ^b^We did not detect lipids in samples with low recovery of nanoparticles (NPs), resulting in a smaller sample size for some lipid species in the NP fractions. For PLA_2_, n = 34, 31, and 18 for CH-NAT, CH-PAT, and AD, respectively.*

We summarize lipid and enzyme changes in our clinical groups (Table 3). For CH-NAT versus CH-PAT, the most significant changes are the increases in SP species in the NP fraction. A lower ceramide to sphingomyelin ratio in the SF fraction and higher ceramide in the NP fraction characterizes CH-NAT versus AD (Table 3). In contrast, we measured higher phosphatidylcholine species and lower ceramide to sphingomyelin ratio in the SF fraction of CH-PAT compared with AD. In the NP fraction, total SPs and three sphingomyelin species are higher in CH-PAT than in AD (Table 3). Both enzyme activities (PLA_2_ and SMase) are similar in the CH groups, but PLA_2_ activity is higher and SMase lower in the AD group than the CH groups (Table 3). Thus, an overall dysregulation of Aβ_42_, tau, GPs, SPs, and lipid enzymes may differentiate the AD biomarker stage from the clinically established dementia stage of AD.

**TABLE 3:** Summary of lipids and enzyme activities changes in cerebrospinal fluid (CSF) fractions.

Changes in the Supernatant Fluid Fraction		
**CH-NAT/CH-PAT**	**CH-NAT/AD**	**CH-PAT/AD**
	The ceramide to sphingomyelin ratio is significantly is lower in CH-NAT than in AD.	PC, PC-SAFA, PC-MUFA, PC34a:0, PC36a:0, and the PC36a:1 levels are higher in CH-PAT than in AD. The ceramide to sphingomyelin ratio is significantly is lower in CH-PAT than in AD.
**Changes in the nanoparticle fraction**		
Total sphingomyelin is lower in CH-NAT than in CH-PAT. SM(35:1), SM(36:2), and the SM(42:3) levels are lower in CH-NAT than in CH-PAT. The ceramide to sphingomyelin ratio is higher in CH-NAT than in CH-PAT.	Ceramide levels are higher in CH-NAT than in AD.	Total sphingomyelin is higher in CH-PAT than in AD. SM(34:2), SM(34:1), and SM(35:1) levels are higher in CH-PAT than in AD.
**Changes in enzyme activities**		
	Acid sphingomyelinase activity and the acid to neutral sphingomyelinase ratio are lower in AD than in CH-NAT.	Phospholipase A_2_ activity is higher in AD than in CH-PAT. Acid sphingomyelinase activity and the acid to neutral sphingomyelinase ratio are lower in AD than in CH-PAT.

### Classification of Study Participants

The study participants who are CH with pathological CSF Aβ_42_/tau have been recently confirmed to be at higher risk for a cognitive decline when we found 11/28 (40%, *p* < 0.0001) of this CH-PAT group declined cognitively with an AD pattern after 4 years, while none of the CH-NAT group had measurable decline (Harrington et al., 2019). We have not studied the CSF of these subgroups after follow-up. However, this CH-PAT group is distinguished from the CH-NAT group by two measures: normal but reduced executive function at baseline (Harrington et al., 2013) and a higher risk for longitudinal decline (Harrington et al., 2019). The CH-PAT individuals are thus a subgroup that has developed CSF biomarkers of AD that precedes symptoms, and cognitive impairment occurs more rapidly in the CH-PAT than in the CH-PAT group.

### GP Dysfunction in the CH-NAT Group

We expected to see lipid changes in neurodegenerative pathology. However, we were surprised to find the different lipid metabolic events at the AD biomarker stage of the CH group compared to the dementia stage. This expansion of GPs in the CSF fluid and membrane fractions reflects a higher turnover of membrane lipids; this may signify an increased propensity for forming inflammatory lipid mediators since the expansion of lipids in cells is consistent with the enhanced formation of inflammatory lipids (Luo et al., 2012; Mirmiran et al., 2014; van Dierendonck et al., 2020). A more dramatic increase in CSF GPs is found in traumatic brain injury, where survival depends on how fast CSF lipids return to normal after injury (Adibhatla and Hatcher, 2008; Pasvogel et al., 2008; Abdullah et al., 2014). A less subtle injury may be in play with CH-PAT study participants, where there is an increase in the release of brain lipids in the CSF. Over the many years of the AD biomarker stage in CH individuals, prolonged release of brain lipids will eventually deplete neuronal cells of vital molecules required for their structure and function. For example, a higher LPC to PC level changes the membrane composition and may indicate altered blood-brain barrier (BBB) transport since LPC species are effectively transported and metabolized across the BBB than unesterified fatty acids (Alberghina et al., 1994; Bernoud et al., 1999).

Other metabolic mechanisms that may lead to the lipid accumulation we report in CH-PAT include changes in uptake, biosynthesis, and lipid remodeling by CoA-dependent and CoA-independent acyltransferases (Chilton et al., 1996; Fonteh et al., 2001). Taken together, our data of the distinct changes in the CH-PAT group suggest that those at risk for AD (CH-PAT) are in a “pro-inflammatory,” “pre-apoptotic” state.

### SP Dysfunction in Preclinical AD

In addition to alterations in GP composition, we show an increase in SM and SM molecular species in CH-PAT compared with CH-NAT. Whereas the increase in PC is mainly in SF, SM’s increase is higher in the NP fraction. These data show that SP metabolism changes can distinguish CH from AD. A subgroup of CH study participants with abnormal CSF proteins similar to AD can be distinguished from AD by their NP-specific increase in SM species.

### Changes in Phospholipases in AD

Several metabolic events may account for the perturbation of CSF lipids in CH-PAT and AD (Chew et al., 2020). GP breakdown by enzymes can alter membrane properties and initiate the formation of inflammatory lipids. For example, PLA_2_ has inflammatory properties and has been shown by immunohistochemical studies to co-localize with amyloid plaques in the brain of AD (Stephenson et al., 1996). We demonstrate that CSF PLA_2_ activity is increased in AD, indicating a role for PLA_2_ in the pathology. It is noteworthy that this increase in AD PLA_2_ activity is not apparent in the CH-PAT stage, nor do the products of PLA_2_ activity (LPC) appreciably change in the CH-PAT stage, which is characterized by the accumulation of lipids including PUFAs in the CSF fractions (Fonteh et al., 2020) and, by inference, in brain cells. The PUFAs are susceptible to oxidation with age that can generate pro-inflammatory lipid products in addition to altering the physical properties of neuronal membranes (Chew et al., 2020). Plasmalogen-specific PLA_2_ that has been reported to play a role in AD (Farooqui and Horrocks, 1998) may account for the depletion of plasmalogen-PE (Senanayake and Goodenowe, 2019; Kling et al., 2020). Examining the major pools of plasmalogen species in CSF, we notice enhanced PE depletion. If PLA_2_ activity is responsible for decreasing plasmalogen (Farooqui and Horrocks, 1998), then there should be an increase in the expression or activation of a plasmalogen-specific PLA_2_ in CSF or within brain cells. Class-specific depletion of lipids coupled with the increase in PLA_2_ product (LPC) in both SF and NP argues for the presence of different enzyme isoforms in CSF. Whereas the decrease in GPs dovetails with the increase in PLA_2_ activity in CSF, there is a paradoxical increase in both PC and LPC in the SF fractions of all three clinical states and mainly in the CH-NAT state for the NP fractions. These data suggest that lipolysis alone does not account for lipid changes in AD. The transport of lipids to a healthy brain, enhanced oxidation in a damaged brain, and increased lipolysis may contribute to lipid abnormalities in preclinical AD (Chew et al., 2020). Thus, the characterization of the different PLA_2_ isoforms in different clinical states, determination of oxidative stress, and the modes of lipid transport into the brain will be fertile areas of future studies to identify mechanisms responsible for plasmalogen depletion in AD.

### The Potential Role of Enzymes in SP Metabolism in the CH-PAT Stage

Examination of SP metabolic pathways suggests that any differences may occur at the synthesis level or via catabolic enzymes such as SMase. Some studies have shown increases in SPMase in AD, and inhibitors of SMase are proposed to have anti-inflammatory and beneficial effects in AD (Chen et al., 2012; Sala et al., 2020). SMase activity was analyzed to determine if the heightened activity was causing elevated CM levels. The assay of neutral SM showed no significant differences in activity among CH and AD. Although SMase activity is the most studied for CM formation, CM may also be derived from secondary pathways such as the breakdown of dhCM by DSE1, the formation of CM by CM synthase, or the breakdown of glucosylceramide by ceramidase. Also, both neutral and acidic SMase is implicated in AD while we have not delineated these activities in our CSF fractions. Acid SMase has recently been shown to influence vesicle formation in glial cells, and several SP metabolic enzymes are associated with Aβ toxicity. We do not expect to detect any of these enzyme changes in CSF if they occur in brain tissues. However, there is evidence in our studies that several of these enzymes are involved in CM formation.

The increase in dhCM can be imparted by enzyme changes also. Several studies show that enhanced oxidative stress can inhibit DSE resulting in the buildup of dhCM in cells (Idkowiak-Baldys et al., 2010; Munoz-Guardiola et al., 2020). Such a process will account for the increase in dhCM in CH-PAT and AD, providing evidence of oxidative stress in preclinical AD. Finally, while the buildup of SM in NP may be due to changes in vesicle formation or “blebbing” during apoptosis of post-mitotic neurons, a decrease in SMase activity and/or an increase in SM synthase may also account for the increase in SM. A precedent for this is provided by the increase in cellular SM in brain diseases due to a defect in SMase activity. Of importance is the fact that such an increase in SM is associated with neuronal degeneration. Could the increase in SM in NP be due to defects in enzyme activities and represent the first signs of neuronal degeneration? We can only glean the answer to this question by characterizing the various enzyme activities in SP metabolism. Additional proteomic studies or genetic knockout studies will tease out the AD defect that accounts for the increase in CM, or in the CH-PAT stage for the increase in SM (NP) or dhCM (SF).

### Implications of GP Changes in AD

What are the implications of altered phospholipid metabolism for the treatment of preclinical pathology and, eventually, AD? Approaches that may reduce abnormal GP metabolism in the CH-PAT stage may include dietary supplementation with omega fatty acids or antioxidants. Though PLA_2_ is not activated in CH-PAT, PLA_2_ activity is increased in AD; therefore, preventing its increase may slow the progression of symptomatic AD. PLA_2_ inhibitors (Farooqui et al., 2006) may help slow the progression of AD in conjunction with modulators of the other known pathologies from Aβ_42_ and oxidative stress. Increased oxidative stress in the presence of higher PUFA concentration in CH-PAT presents a unique environment for the formation of oxidized lipid products with inflammatory or neurotoxic properties. Abrogation of these early biochemical events has the potential for preventing neuronal cell death and thus preventing disease transition from the CH-PAT to the AD stage.

### Regulating SP Metabolism in Preclinical AD –Changes in SP Metabolism in CH-PAT and AD

The major SPs we detect in CSF fractions are sphingomyelin (SM), ceramide (CM), and dihydroceramide (dhCM; Fonteh et al., 2015). SM is formed by SM synthase, dhCM is synthesized by dhCM synthase. CM can be formed from SM by one of three different SMase (neutral SMase, acid SMase, and alkSMase), *de novo* synthesis, or using sphingosine and a fatty reacylation. In the NP fraction from CH-PAT, there is an increase in SM and dhCM. The increase in SM may be accounted for by an increase in SM synthase and/or a decrease in SMase activities. Alternatively, defects in autophagogocytosis can result in the abnormal breakdown of membrane lipids with a subsequent increase of SM in NP.

Similarly, the increase in dhCM levels in NP from CH-PAT may be accounted for by increasing dhCM synthase and/or decreasing dhCM desaturase. In contrast to CH-PAT, there is an increase in CM in AD. An increase in CM may be due to an increase in the SMase activity or the other enzyme pathways that form CM via *de novo* synthesis or the Salvage pathway. The net effect of these enzymatic changes is the alteration in SP metabolism resulting in neuronal dysfunction. At the center of our data is the enhanced breakdown of SM in SF for AD shown by a significant increase in the CM/SM ratio. This increase accompanies an increase in SM and total SP in the preclinical samples (CH-PAT). These data suggest that compounds that alter SM breakdown can be useful in AD. An example is minocycline, an anti-inflammatory compound, an inhibitor of SMase activity reported to be a useful agent for controlling the progression of AD (Chen et al., 2012). If an increase in apoptosis of neurons accounts for the increase in SM in NP, then agents that prevent this process can also be monitored. Known small molecule enhancers of autophagocytosis affect lipid signaling and have the potential beneficial effects on AD. Thus, our study identified a subset of CH subjects with abnormal amyloid/tau and skewed SM metabolism that may benefit from this type of intervention.

### Study Limitations

Our study has significant implications for GP and SP regulation in aging. However, it is limited by the cross-sectional design and by the fact that there is no a *priori* study that we could use to design our study better. The clinical and CSF biomarker classification of our groups is based on neuropsychology and measures of CSF Aβ_42_/tau, and we are still establishing an association of our classification with the conventional NIA/AA (ATN) trajectories (Tan et al., 2020). We did not control for dietary intake of lipids and do not know how medications and other confounders (genetics, age, gender, race) may affect brain lipids. We attempt to mask any interference by these confounders by matching them in our clinical groups (Harrington et al., 2013; Fonteh et al., 2020). The preparation of the SF and NP fractions involves complex fractionation using differential centrifugation. The analyses of complex lipids involve manipulations that may introduce errors in our analyses. None the less, we have attempted to normalize our data using internal standards for the most accurate quantification of CSF lipids.

## Conclusion

There is a pressing need for sensitive biomarkers for early diagnosis and monitoring treatments of AD. The CH-PAT and AD have similar Aβ_42_/tau pathology; however, GP and SP alterations in the CSF can distinguish these groups. GPs and SPs differences also distinguish the CH-PAT from CH-NAT biomarker CH groups. Therefore, the measured phospholipid composition in CSF fractions not only indicates a mechanistic disturbance in the CH-PAT stage but can be combined with Aβ_42_/Tau as a biomarker of early AD pathology. The increase in SM species in NP opens another exciting area of research to determine whether the CH-PAT cohort with increased SM in NP undergoes a faster transition to AD and test whether agents that enhance phagocytosis (Roig-Molina et al., 2020) may prevent modifications in SP metabolism. Finally, the increase in dhCM in CH with abnormal proteins points to oxidative stress in the CH-PAT group. It may be useful in monitoring the efficacy of antioxidants in preventing the onset of AD. Overall, our data reveal striking changes in GP and SP metabolism in a CH group distinguished with AD CSF biomarkers that can motivate the development of early detection methods and therapies to lessen lipid imbalances in the complex pathophysiology of AD.

## Data Availability Statement

The raw data supporting the conclusions of this article will be made available by the authors, without undue reservation.

## Ethics Statement

The studies involving human participants were reviewed and approved by Huntington Memorial Hospital, Pasadena. The patients/participants provided their written informed consent to participate in this study.

## Author Contributions

AF and MH contributed to the conceptualization and study design, writing of original draft and manuscript preparation, project administration, resources, and funding acquisition. AF, AC, SE, and MH contributed to the methodology. AF contributed to validation and supervision and the formal data analyses. AC, MH, SE, and AF contributed to the data curation. XA, MH, and AF contributed to the expert manuscript review and editing. All authors contributed to the article and approved the submitted version.

## Conflict of Interest

The authors declare that the research was conducted in the absence of any commercial or financial relationships that could be construed as a potential conflict of interest.

## Footnotes

1 www.lipidmaps.org
